# Prenatal diagnosis of cranio-orbito-maxillary neuroblastoma: a case report

**DOI:** 10.1515/crpm-2025-0030

**Published:** 2026-09-25

**Authors:** Busra Berfin Polat, Rauf Melekoglu, Servet Guresci

**Affiliations:** Faculty of Medicine, Department of Obstetrics and Gynecology, Inonu University, Malatya, Türkiye; Ankara Bilkent City Hospital, Department of Pathology, Ankara, Türkiye

**Keywords:** case management, fetus, neuroblastoma, orbital neoplasms, prenatal diagnosis, prenatal ultrasonography

## Abstract

**Objectives:**

Congenital neuroblastoma is one of the most common solid tumors in the perinatal period; however, primary cranial localization with prenatal onset is extremely rare. We present a unique case of cranio-orbito-maxillary congenital neuroblastoma detected during the early second trimester, highlighting its dynamic prenatal evolution, diagnostic challenges, and postnatal clinical course.

**Case presentation:**

A 36-year-old pregnant woman was referred at 15 weeks of gestation with a suspected fetal cranial cyst. Fetal neurosonography revealed a unilocular, avascular cystic lesion near the midline of the left cerebral hemisphere, initially suggestive of an arachnoid cyst. Serial follow-up demonstrated progressive morphological transformation, with the lesion acquiring heterogeneous solid components and extending toward the retroorbital and maxillary regions. Fetal magnetic resonance imaging confirmed a large solid-cystic retroorbital mass with orbital displacement and extension into adjacent structures. The pregnancy was complicated by polyhydramnios in the third trimester. A female infant was delivered at 36 weeks of gestation and exhibited marked postnatal proptosis. Histopathological and immunohistochemical evaluation of a tru-cut biopsy established the diagnosis of grade 4 neuroblastoma. Despite multidisciplinary management and multimodal chemotherapy, the disease progressed with pulmonary metastases, and the infant died at 15 months of age during palliative care.

**Conclusions:**

This case illustrates an exceptionally rare presentation of congenital neuroblastoma originating as a prenatal cranial cystic lesion with progressive cranio-orbito-maxillary involvement. Neuroblastoma should be considered in the differential diagnosis of atypical fetal cranial or orbital masses. Careful prenatal surveillance, advanced imaging, and multidisciplinary perinatal planning are crucial for optimal postnatal management and family counselling.

## Introduction

Neuroblastoma is one of the most common solid tumors in the perinatal period and constitutes a broad spectrum of neuroendocrine tumors originating from neural crest progenitor cells. During embryogenesis, the migration of neural crest cells from their original locations to settle around embryonic vascular structures is critical in neuroblastoma biology because signal transmission abnormalities that arise during this process can pave the way for uncontrolled proliferation and tumor transformation. This tumor spectrum includes neuroblastoma, ganglioneuroblastoma, and ganglioneuroma, and the literature clearly reports that it exhibits significant heterogeneity in terms of biological behavior [1], [2], [3]. Congenital or fetal neuroblastomas may present clinically, radiologically, and prognostically significant differences from cases detected later in childhood [4]. These cases are mostly detected incidentally during prenatal ultrasound or fetal magnetic resonance imaging [4]. Neuroblastoma accounts for approximately 10 % of childhood tumors but is responsible for approximately 15 % of childhood tumor-related deaths. Prenatal diagnosis is critically important for predicting the tumor’s biological behavior, planning perinatal management, and determining the postnatal treatment approach [4], 5]. The incidence of fetal neuroblastoma has been reported in different series to be between 1/10,000 and 1/100,000 live births [5]. Owing to the migratory potential of neural crest cells, tumors can exhibit a wide anatomical distribution spectrum. However, it is most commonly observed in the thoracic, cervical, abdominal, and pelvic regions [4], 5]. Most extracranial neuroblastomas are adrenal in origin; indeed, approximately half of fetal or pediatric adrenal masses are reported to be neuroblastomas [4]. Therefore, solid masses identified in the adrenal region during the prenatal period provide important diagnostic clues [4]. Clinically, neuroblastomas are often detected as solid masses; presentation in the form of cystic lesions is quite rare, and only a limited number of cases have been reported in the literature [5], 6]. Neuroblastomas diagnosed during the fetal period are usually primarily extracranial; metastatic spread to the cranial region may occur, but more rarely, the disease may be primarily cranial. Primary cranial neuroblastomas are quite rare and account for only approximately 8 % of reported cases [6], 7]. The case we present is a rare example of a cranio-orbito-maxillary congenital neuroblastoma that initially appeared as a cystic lesion in the cranial region during the early second trimester; progressed over time toward the orbit and maxilla; acquired solid components; and was diagnosed clinically, radiologically, and histopathologically. The anatomical spread pattern of the lesion, the morphological transformation it underwent during the prenatal period, and its postnatal confirmation represent a highly noteworthy clinical picture that is only sparsely described in the literature [7].

## Case presentation

**Figure 1:**
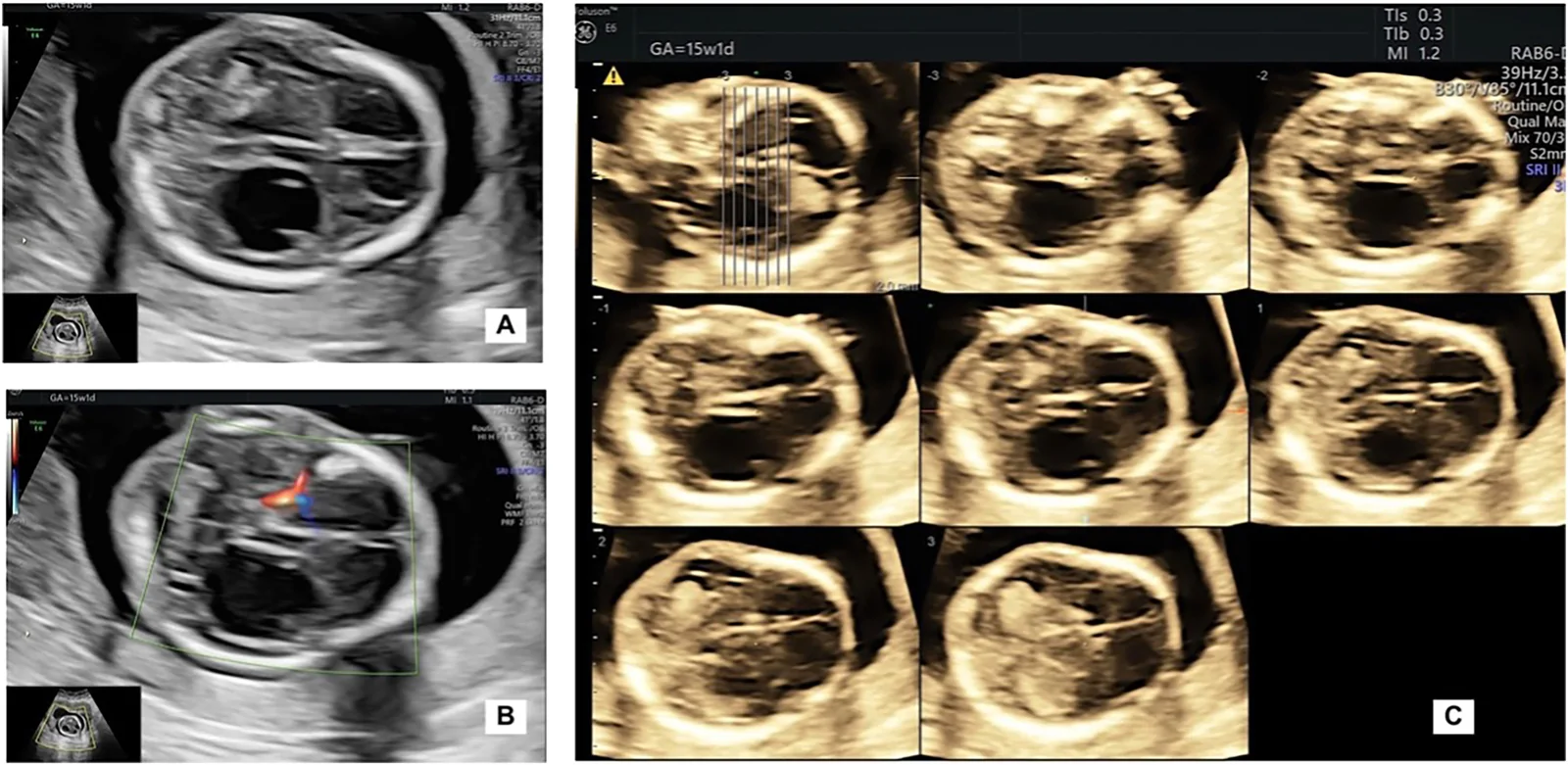
Fetal neurosonography findings at 15 weeks of gestation. (A) Unilocular, anechoic cystic structure in the axial plane, close to the midline in the left hemisphere, unrelated to the lateral ventricles. (B) Demonstration of the avascular character of the same lesion using color Doppler. (C) Appearance of the unilocular structure and intracranial location of the cystic lesion in multiple sections.

A 36-year-old patient, gravida 4 para 3, with no congenital anomalies in previous pregnancies and no family history of hereditary disease, who was assessed as “low risk” during the first trimester screening on the basis of the combined test results, was referred to us at 15 weeks of gestation with a preliminary diagnosis of a fetal cranial cyst. Fetal sonographic evaluation revealed a single pregnancy with a positive fetal heartbeat, biometric measurements consistent with gestational age, and a normal amniotic fluid volume. Fetal neurosonography revealed a unilocular, avascular cystic lesion measuring 17 × 13 mm in the axial plane near the midline and 16 × 14 mm in the sagittal plane in the left cerebral hemisphere, unrelated to the lateral ventricles (Figure 1). Given its morphological features, an arachnoid cyst was considered the primary diagnosis. The family was informed that arachnoid cysts usually occur in isolation but may rarely be associated with corpus callosum agenesis and chromosomal abnormalities, particularly trisomy 18. It was explained that neurodevelopment generally proceeds normally in isolated small cysts; however, large cysts or those causing compression may require surgical intervention during the neonatal period and may lead to long-term sequelae such as epilepsy and motor delay.

**Figure 2:**
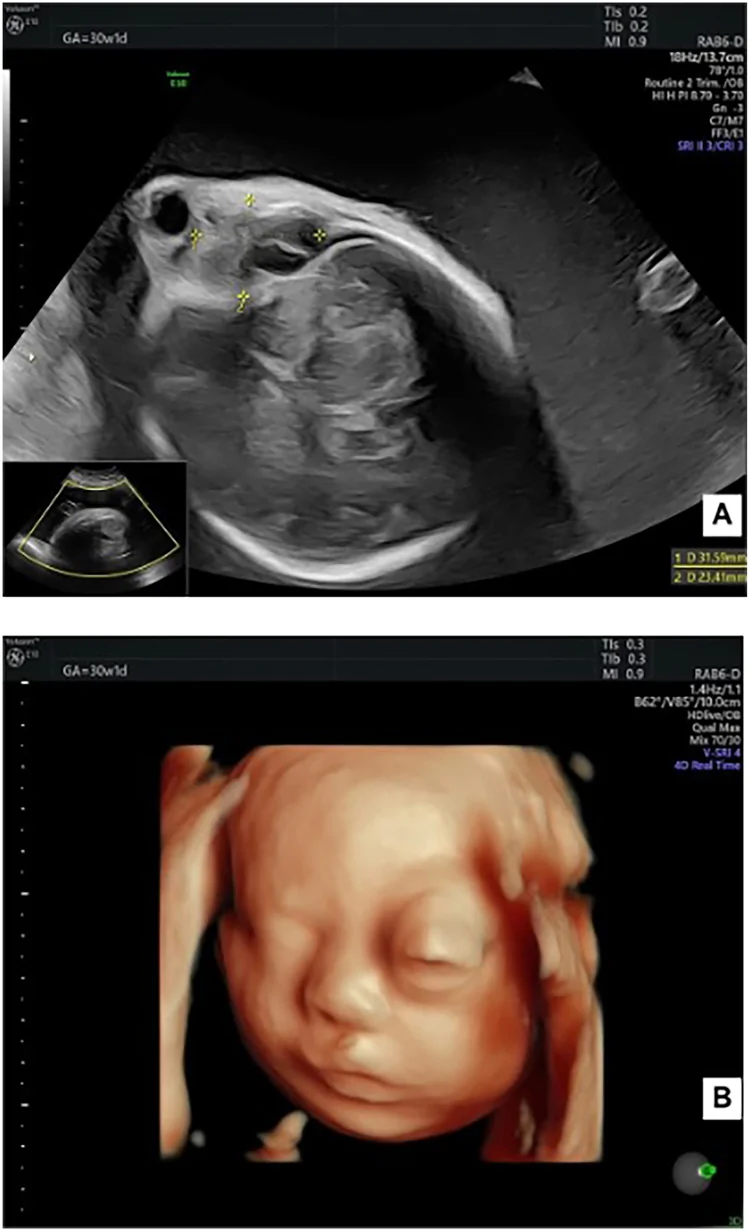
Fetal ultrasound findings at 30 weeks of gestation. (A) A solid-cystic mass extending toward the left retroorbital area, with heterogeneous content. (B) Three-dimensional image of the anterior compression of the facial region caused by the same lesion, as seen on 4D ultrasound.

**Figure 3:**
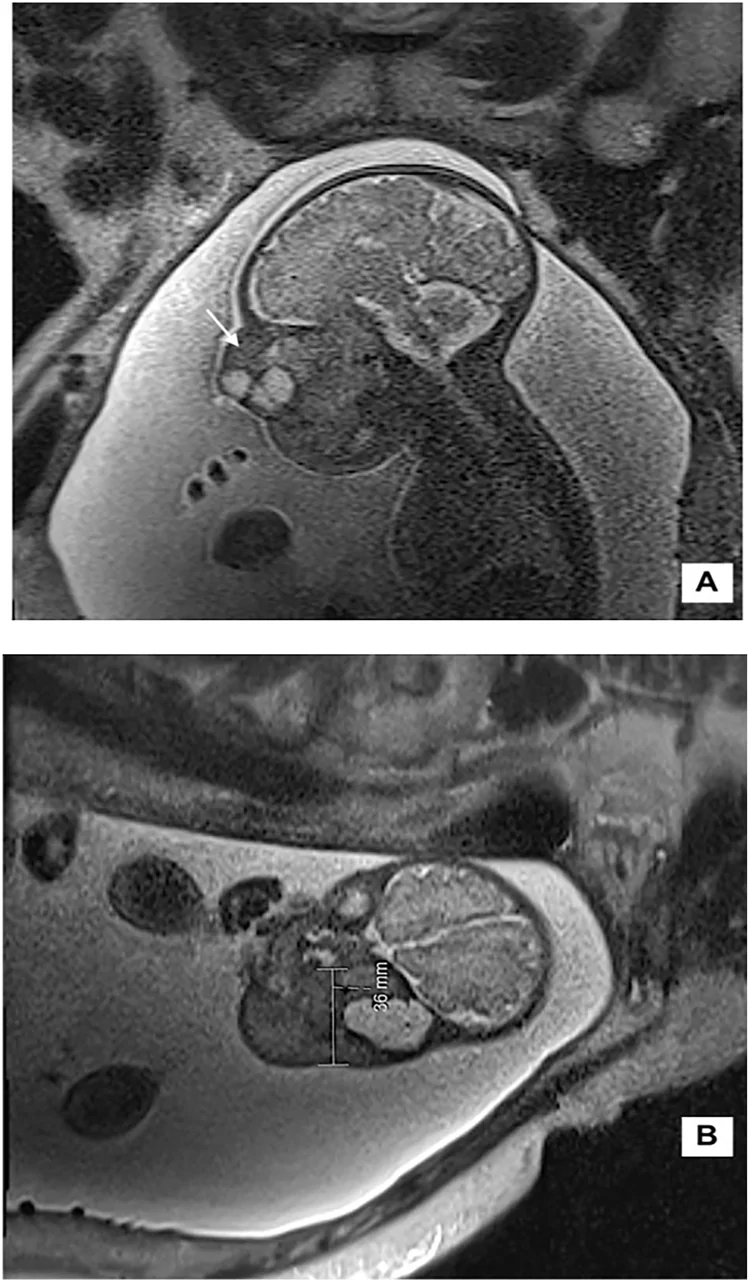
Fetal magnetic resonance imaging findings. (A) Mass (indicated by the arrow) located in the left retroorbital space, pushing the globe anteriorly, with a solid-cystic internal structure. (B) Sagittal section showing the lesion’s extension into the retropharyngeal space and its approximately 6 cm craniocaudal dimension.

The family was also informed that a normal combined test does not completely rule out chromosomal abnormalities and that options for further evaluation, such as cell-free fetal DNA testing in maternal blood and invasive prenatal diagnosis (amniocentesis), are available; however, the family declined further genetic screening and diagnostic methods. The option of pregnancy termination was also presented to the family during the initial evaluation, but the family expressed their determination to continue the pregnancy. Additionally, it was noted that advanced evaluation could be performed via fetal cranial magnetic resonance imaging (MRI) in the third trimester. During the patient’s subsequent follow-up examinations, the lesion, initially observed as an anechoic cystic structure, progressively acquired heterogeneous content and progressed toward the orbital region. The family was informed about this change, but no additional fetal anomalies were detected. Polyhydramnios was observed via ultrasound at 30 weeks of gestation. On fetal neurosonography, the lesion previously identified in the left parietal region, which was initially anechoic and cystic in character, was observed to have acquired a distinctly heterogeneous content, reached a size of approximately 31 × 23 mm, and extended toward the left retroorbital area. Fetal MRI was planned because of the enhancement of the solid components and the progression of the lesion (Figure 2). Fetal MRI revealed a mass located in the left retroorbital region, prominently displacing the globe anteriorly, measuring approximately 6 × 3.5 × 6 cm, with lobulated contours and containing solid and cystic areas. The lesion was assessed to extend inferiorly toward the retropharyngeal space, with a possible thin transition zone to the intracranial cavity but no evidence of significant intracranial invasion (Figure 3).

**Figure 4:**
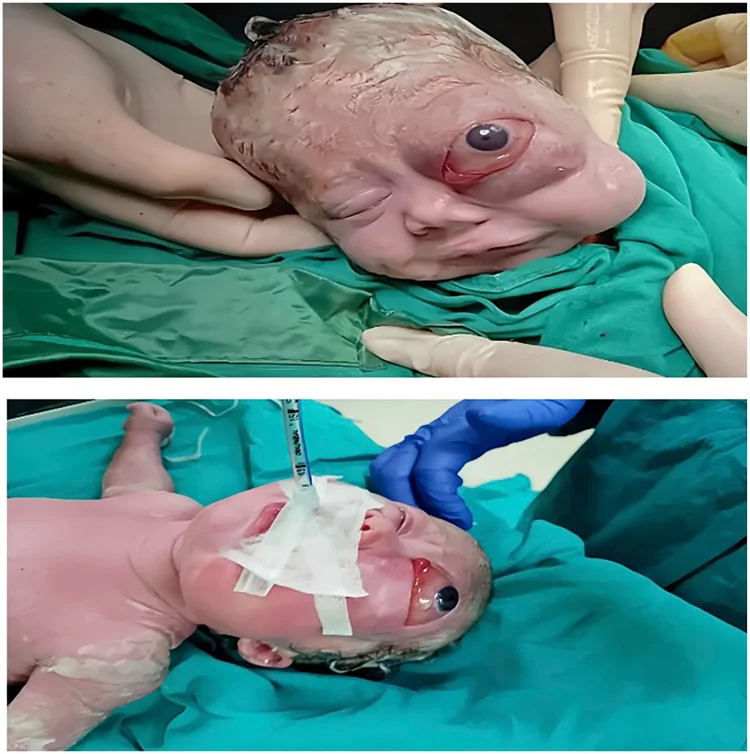
Postnatal evaluation findings. The first postnatal examination revealed marked proptosis in the left orbit, which is attributed to the anterior pressure of the retroorbital solid-cystic mass identified during the fetal period. The extension of the tumor tissue to the maxillary region is evident from the facial asymmetry.

The extension of the tumor into the orbital region, its heterogeneous and solid characteristics, and the minimal increase in size observed in serial follow-up examinations revealed that the likelihood of malignancy had increased. In the clinical differential diagnosis, rhabdomyosarcoma, teratoma, Ewing sarcoma, hemangioma, myoblastoma, and neuroblastoma were considered the primary possibilities. No additional pathology other than polyhydramnios developing in the third trimester was detected during pregnancy. The multidisciplinary council assessment was planned for postnatal management to be carried out in coordination with the pediatric neurology, ophthalmology, and pediatric neurosurgery teams. The family was given detailed counselling on the possible postnatal process, surgery, and treatment requirements, and routine pregnancy follow-up visits were continued. At 36 weeks of gestation, the patient underwent a cesarean section due to premature rupture of membranes. A female infant weighing 2,500 g with an APGAR score of 7/8 was delivered. During the postnatal period, while the infant was being intubated due to respiratory distress, marked proptosis was observed due to a tumor mass located in the left retroorbital region (Figure 4). No additional congenital anomalies were detected during the initial evaluation. The newborn was admitted to the neonatal intensive care unit for further evaluation and stabilization.

**Figure 5:**
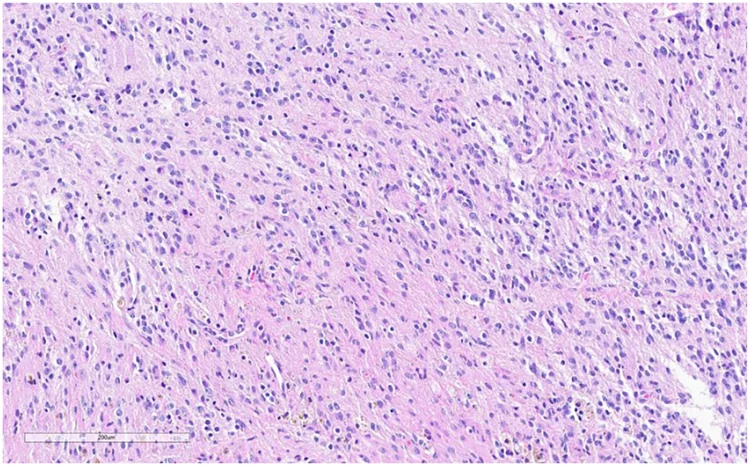
Tumor morphology under hematoxylin-eosin (H&E) staining. A typical neuroblastic proliferation consisting of small round cells with a high nucleus/cytoplasm ratio showing diffuse infiltration on a fibrillar neuropil background is observed (20 × magnification).

**Figure 6:**
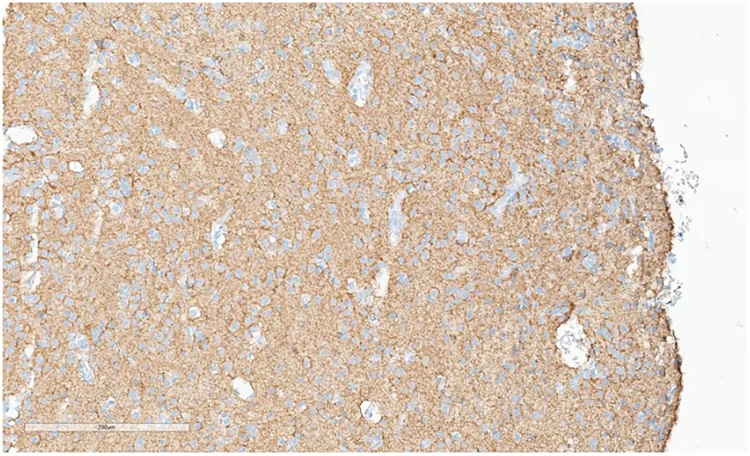
Immunohistochemical staining for synaptophysin. Significant cytoplasmic synaptophysin positivity is observed in tumor cells, supporting neuroendocrine differentiation. This finding, together with histomorphology, strengthens the diagnosis of neuroblastoma (20 × magnification).

Findings detected on postnatal orbitocranial MRI were consistent with the prenatal period, and the patient’s oncological markers, including catecholamine metabolites, were reported within normal limits for her age. A tru-cut biopsy was performed on the 17th postnatal day for the mass in the left orbital region. Histopathological examination revealed a typical neuroblastic tumor morphology consisting of small round cells with a high nucleus/cytoplasm ratio and diffuse infiltration on a fibrillar neuropil background. The immunohistochemical profile of the tumor cells revealed widespread positivity for synaptophysin and Olig2, supporting neuroendocrine and neuroblastic differentiation. The negativity of CD99, vimentin, GFAP, and desmin stains helped exclude other small round cell tumors considered in the differential diagnosis. INI-1 expression was found to be preserved. The Ki-67 proliferation index was calculated as 8–10 % in “hot-spot” areas. When all histomorphological and immunophenotypic findings were evaluated together, the lesion was reported to be consistent with Grade 4 neuroblastoma according to the World Health Organization (WHO) classification (Figures 5 and 6).

The patient’s treatment process was evaluated at regular intervals by a multidisciplinary team (ophthalmology, pediatric oncology, neonatal intensive care). Postnatal chest imaging revealed multiple parenchymal lesions measuring 20 × 10 mm in the posterobasal segment of the right lower lobe and 14 × 8 mm in the superior segment of the left lower lobe, with surrounding atelectatic and fibrotic retractions, some cystic-cavitary, some pure solid, and irregularly bordered lesions. These findings were interpreted as pulmonary metastases from a primary tumor located in the retroorbital region. Considering the patient’s age, weight status, and overall clinical stability, initiating neoadjuvant chemotherapy was deemed appropriate. Within this scope, the first cycle consisted of vincristine–actinomycin D; the second, third, and fourth cycles consisted of carboplatin–etoposide (with a 30 % dose reduction depending on age and condition); and the fifth and sixth cycles consisted of the vincristine–temozolomide–irinotecan (VIT) regimen (fifth cycle: 50 % dose; 6th cycle: full dose). Early-stage imaging revealed slight progression in the size of the mass (from 8.3 × 4.2 × 8.4 cm to 8.5 × 4.5 × 8.7 cm), and treatment was planned to continue at full dose with the same regimen. However, chemotherapy was discontinued due to the development of sepsis on the third day of the patient’s 7th cycle of VIT therapy. In the subsequent period, it was decided that continuing systemic intravenous chemotherapy was not appropriate due to recurrent episodes of sepsis. Following multidisciplinary re-evaluation, it was recommended that systemic treatment be completely discontinued. Although the council recommended palliative radiotherapy, the family did not accept this option. Following a complication of intracranial hemorrhage that developed during the palliative care process, the patient died 15 months after birth.

## Discussion

Although congenital neuroblastomas are among the most common solid tumors detected during the perinatal period, cases with primary cranial localization have been reported extremely rarely in the literature [5], [6], [7]. Cases originating in the cranial region and exhibiting a progressive spread pattern toward the orbitomaxillary structures are even rarer. This situation highlights the biological heterogeneity of neuroblastomas and the decisive role of the differentiation and migration patterns of neural crest-derived cells in tumor development. The unusual progression observed in our patient, which originated in the cranium and subsequently extended to the orbitomaxillary compartments, suggests that congenital neuroblastomas form a broad spectrum not only in terms of anatomical localization but also in terms of their developmental origins and growth dynamics. The vast majority of congenital neuroblastomas are adrenal in origin, and cases showing primary cranial or orbital localization are extremely rare [4], [5], [6]. A significant proportion of the few orbital neuroblastomas described in the literature are associated with secondary invasion of surrounding tissues or metastatic spread from the primary tumor [6], 7]. Cystic neuroblastomas constitute only a small proportion of all cases and generally develop due to secondary changes such as necrosis, hemorrhage, or spontaneous regression during the tumor differentiation process [8]. Therefore, the presence of a lesion that begins as cystic in nature during the prenatal period and acquires solid components over time, as in our case, is a noteworthy finding reflecting the dynamic and evolutionary biological structure of congenital neuroblastomas [8], 9]. Since cystic cranio-orbital masses detected during the prenatal period can often be confused with other congenital lesions, such as teratomas, cystic hygromas, craniopharyngiomas, or dermoid cysts, advanced imaging methods that allow tissue characterization – especially fetal and postnatal MRI – play a critical role in differential diagnosis [8]. Orbital and maxillary neuroblastomas may present clinically with proptosis, periorbital edema, nasal obstruction, or maxillofacial asymmetry [8]. The definitive diagnosis is established by integrating prenatal imaging findings with postnatal radiological evaluation and histopathological confirmation [8], 9]. Furthermore, genetic alterations such as MYCN (MYCN proto-oncogene, bHLH transcription factor) amplification are known to be important prognostic markers for determining the biological behavior of tumors [8], 10]. In the present case, the tumor’s cranial origin and progression toward the orbit and maxilla suggest both a possible variation in the embryonic migration of neural crest cells and local invasive growth potential. The transformation from a cystic lesion to a solid structure may reflect biological dynamics such as differentiation, necrosis, or hemorrhagic remodelling that occur during the maturation processes of neuroblastomas [9]. Although isolated orbital neuroblastomas have been reported in the literature, the pattern of spread observed in the present case, which originated in the cranium and extended to the orbitomaxillary compartments, represents a rare variant of this tumor group. The treatment approach varies depending on the tumor stage, biological risk profile, genetic characteristics, and feasibility of surgical resection. While surgical excision alone may be sufficient for congenital and localized lesions, multimodal treatment and adjuvant chemotherapy may be necessary in patients with high-risk biological characteristics or those showing spread. Therefore, integrating a multidisciplinary approach in the postnatal period with early prenatal diagnosis and regular long-term follow-up is highly important for improving the prognosis of such rare cases of cranio-orbito-maxillary neuroblastoma [10].

## Conclusions

Fetal retroorbital tumors are extremely rare, and diagnosis during the prenatal period and early planning of a multidisciplinary approach significantly improve postnatal management. The presented case represents an exceptionally rare clinical presentation in the literature, as it was detected during the prenatal period, initially presented as a cystic lesion in the cranial region, progressed to involve the orbit and maxilla with a solid character in subsequent weeks, was classified as stage 4 in the postnatal histopathological examination, and had lung metastases. In the literature, no similar case has been reported in which neuroblastomas, which begin as cranial cystic lesions and progress toward orbital–maxillofacial regions, were detected during the prenatal period. This situation highlights how important prenatal diagnosis is in terms of postnatal preparation, treatment planning, and family counselling. In conclusion, this case underscores the importance of considering neuroblastoma among the differential diagnoses of atypical head and facial masses detected in the fetal or neonatal period, highlighting the value of multidisciplinary management and informed family counselling. The inclusion of the presented case in the literature will provide valuable contributions in terms of encouraging the reporting of similar cases and better understanding the clinical course, biological behavior, and treatment strategies of these tumors. Furthermore, this case highlights the critical importance of regular and meticulous prenatal monitoring, timely advanced imaging, and clinician experience in identifying lesions that can be confused with benign lesions such as arachnoid cysts, particularly during the prenatal period, but that can acquire a solid character during follow-up and transform into a completely different pathology.
